# Supplementary figures and images for: Mapping and Dynamics of Regulatory DNA in Maturing Arabidopsis thaliana Siliques
Source: Front Plant Sci. 2019 Nov 14;10:1434. doi: 10.3389/fpls.2019.01434 (PMC6868056; doi:10.3389/fpls.2019.01434)

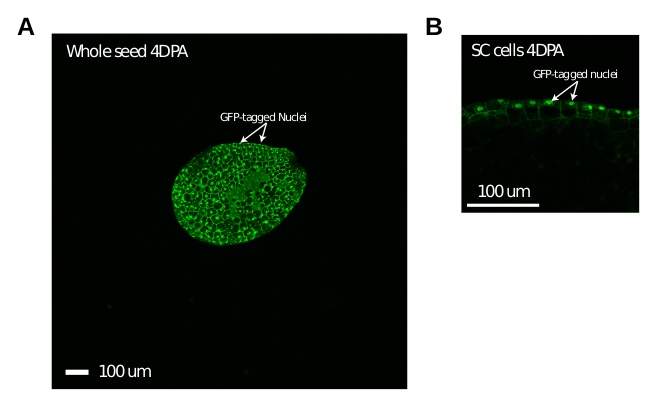

Supplement: Supplemental Figure 1 — Confocal microscopy of INTACT-tagged nuclei in seed coat epidermis. (A) Confocal of whole seed at 4DPA from the INTACT line GL2pro:NTF/ACT2pro:BirA (Deal and Henikoff, 2010). GFP-fluorescing nuclei are evident across the seed coat epidermis. Scale is 100um. (B) Confocal of 4DPA mucous secreting cells (MSCs) from the INTACT line GL2pro:NTF/ACT2pro:BirA (Deal and Henikoff, 2010). GFP-fluorescing nuclei are readily observable in the outer most layer of the seed coat. Scale is 100 um. [file Image_1.jpeg]

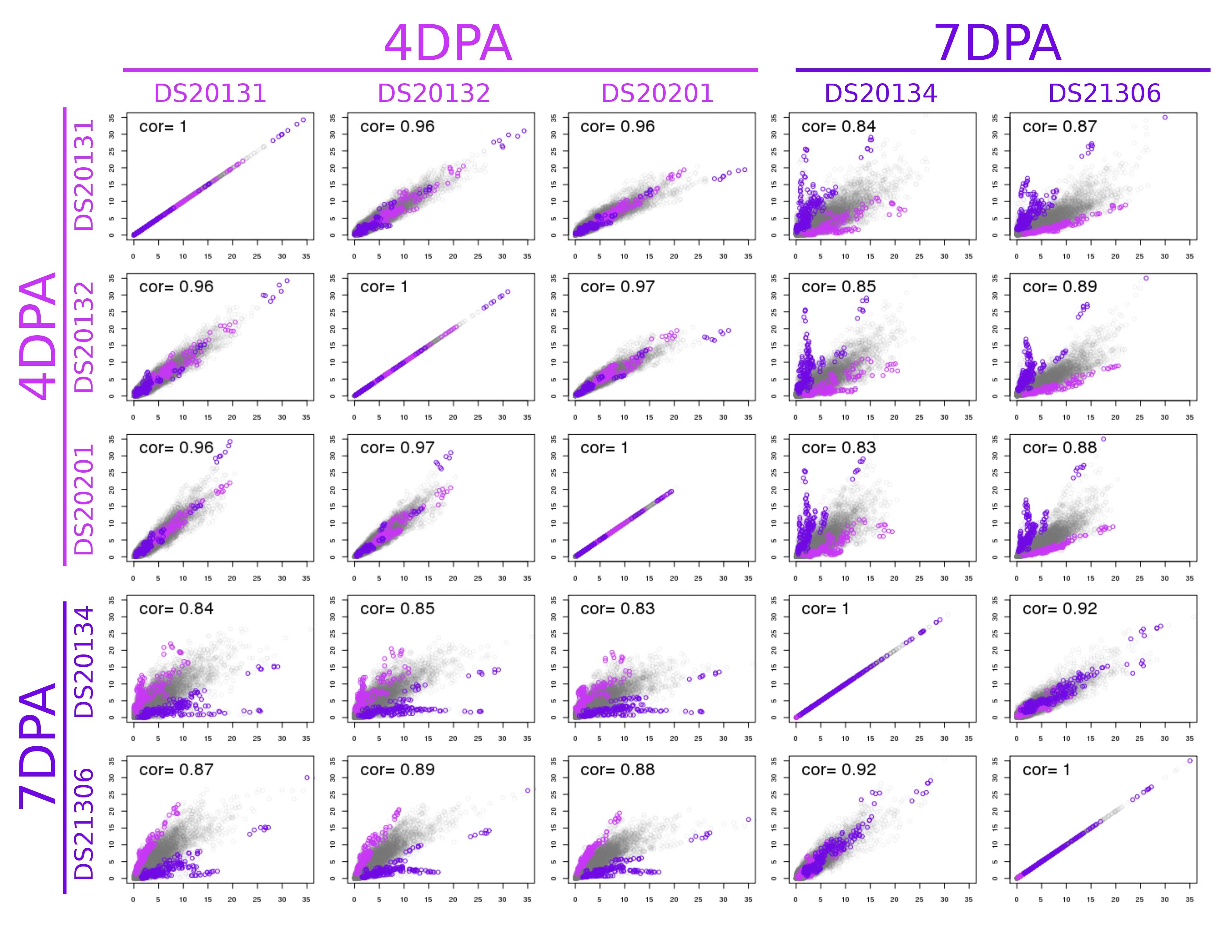

Supplement: Supplemental Figure 2 — Correlation of normalized cut counts within 150bp windows. The three replicates of one time point (4DPA) had pairwise Pearson's correlation coefficients (PCCs) of 0.96 and 0.97. The two replicates of the other time point (7DPA) had a 0.92 Pearson's correlation coefficient. PCCs between time points were lower (maximum PCC=0.89), with multiple obvious outliers corresponding to regions of differential accessibility (light purple: regions identified in subsequent analysis as more accessible in 4DPA; dark purple: regions identified in subsequent analysis as more accessible in 7DPA). Plots display the normalized cut counts within 100,000 150 bp sliding windows (130 bp overlap) in the first 2,000,045 bp of chromosome 1. [file Image_2.jpeg]

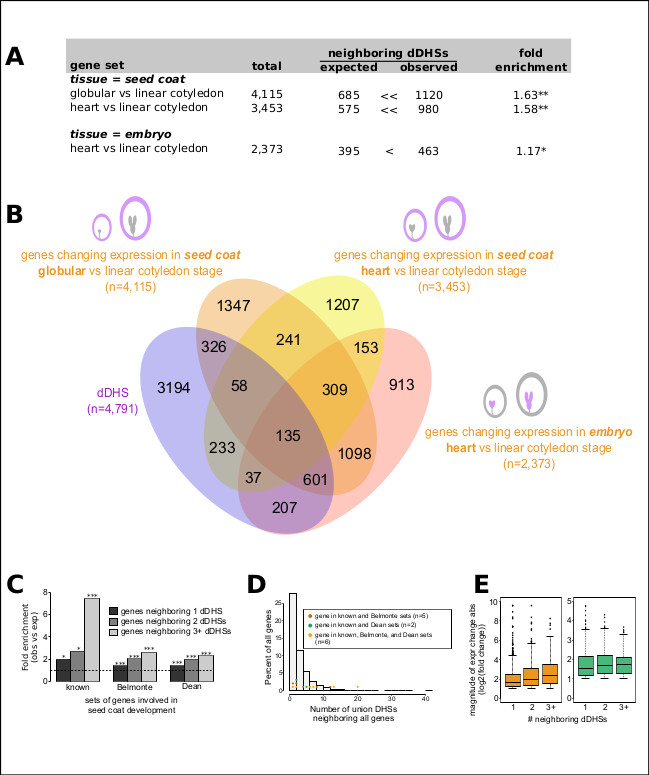

Supplement: Supplemental Figure 3 — Genes neighboring developmentally dynamic DHSs are often differentially expressed in seed coat and embryo. (A) Overlap between the set of genes neighboring dDHSs and genes differentially expressed in seed coat at globular vs linear cotyledon stage and heart vs linear cotyledon stage, and genes differentially expressed in embryo at heart vs linear cotyledon stage (Belmonte et al. 2013). One asterisk (*) indicates p-value < 0.01. Two asterisks (**) indicate p-value < 10-20. (B) Overlap of all four sets of genes. (C) The set of genes neighboring multiple dynamic DHSs tend to contain more genes related to seed coat development than expected at random. This is seen in the set of 59 known seed coat development genes ( Supplemental Table 1 ) as well as in genes with differential expression (Dean et al. 2011; Belmonte et al. 2013). Dashed line indicates Fold Enrichment of 1, which indicates no enrichment over random expectation. Significance of difference in Fold Enrichment compared to random expectation indicated by asterisks: *p-value<0.05, ***p-value<1e6. (D) Distribution of number of union DHSs neighboring all genes. The number of union DHSs neighboring the 13 known genes that are differentially expressed in the Belmonte and/or Dean set but did not neighbor dynamic DHSs are indicated by the placement of colored dots along the x-axis. Of these 13 genes, five were identified only in the Belmonte set (AT1G79840, AT3G13540, AT5G67360, AT2G37260, and AT1G21070), two only in the Dean set (AT5G67030 and AT3G09820), and six in both sets (AT5G23940, AT1G02720, AT4G36890, AT3G15510, AT2G18280, and AT5G35550). (E) The magnitude of the change in expression of genes neighboring 1, 2, and 3-or-more dynamic DHSs. Orange boxplots show expression change in the Belmonte et al. 2013 data set. Green boxplots show expression changes in the Dean et al. 2011 data set. The difference in mean between the abs(log2(fold change)) for genes neighboring 2 and 3-or-more dynamic DHSs and those neighbo [file Image_3.jpeg]
